# A multi-trap microfluidic chip enabling longitudinal studies of nerve regeneration in *Caenorhabditis elegans*

**DOI:** 10.1038/s41598-017-10302-4

**Published:** 2017-08-29

**Authors:** Sertan Kutal Gokce, Evan Marley Hegarty, Sudip Mondal, Peisen Zhao, Navid Ghorashian, Massimo A. Hilliard, Adela Ben-Yakar

**Affiliations:** 1 Electrical and Computer Engineering, The University of Texas at Austin, Austin, TX 78712 USA; 2 Mechanical Engineering, The University of Texas at Austin, Austin, TX 78712 USA; 3 Biomedical Engineering, The University of Texas at Austin, Austin, TX 78712 USA; 4 Queensland Brain Institute, The University of Queensland, Brisbane, QLD 4072 Australia; 5 Institute for Neuroscience, The University of Texas at Austin, Austin, TX 78712 USA

## Abstract

Several sophisticated microfluidic devices have recently been proposed for femtosecond laser axotomy in the nematode *C. elegans* for immobilization of the animals for surgery to overcome time-consuming and labor-intensive manual processes. However, nerve regeneration studies require long-term recovery of the animals and multiple imaging sessions to observe the regeneration capabilities of their axons post-injury. Here we present a simple, multi-trap device, consisting of a single PDMS (polydimethylsiloxane) layer, which can immobilize up to 20 animals at the favorable orientation for optical access needed for precise laser surgery and high-resolution imaging. The new device, named “*worm hospital”* allows us to perform the entire nerve regeneration studies, including on-chip axotomy, post-surgery housing for recovery, and post-recovery imaging all on one microfluidic chip. Utilizing the worm hospital and analysis of mutants, we observed that most but not all neurodevelopmental genes in the Wnt/Frizzled pathway are important for regeneration of the two touch receptor neurons ALM and PLM. Using our new chip, we observed that the *cwn-2* and *cfz-2* mutations significantly reduced the reconnection possibilities of both neurons without any significant reduction in the regrowth lengths of the severed axons. We observed a similar regeneration phenotype with *cwn-1* mutation in ALM neurons only.

## Introduction

The capacity of neurons, especially those in the adult mammalian central nervous system, to regenerate after injury is limited, leading to permanent deficits in the nervous system^1, 2^. A thorough exploration and understanding of the mechanisms behind nerve regeneration are important for developing novel and efficient therapeutics for acute trauma, spinal cord injuries, and other neurodegenerative diseases. The nematode *Caenorhabditis elegans* has become a popular model organism for the study of nerve regeneration at the cellular and molecular level because of its many favorable properties. It has a transparent body that allows for optical observation of individual neurons, as well as short lifespan and generation time, evolutionarily conserved nerve regeneration mechanisms, and amenability to *in vivo* axotomy using ultrafast laser pulses. These unique features make *C. elegans* an ideal model for large-scale genetic and drug screenings. Our pioneering use of femtosecond laser axotomy to investigate the regeneration capacity of GABAergic motor neurons in *C. elegans* ^3^ has driven many other labs to use this approach to study the molecular bases of nerve regeneration *in vivo* in a number of other *C. elegans* neurons^4–14^.

A precise laser targeting of 300-nm diameter axons requires co-localizing the axon of interest with the focal volume of the laser beam with a high spatial accuracy, and thus a complete immobilization of the nematode during the axotomy procedure. Immobilization methods using anesthetics^9, 14, 15^ or polystyrene beads^16, 17^ necessitate manually orienting animals to get the neuron of interest near the glass cover slip interface. These tasks must be accomplished without losing or harming the animals during their transfer from anesthetics or polystyrene beads via liquid buffer exchange^3, 16^. Moreover, the animals need to be transferred from nematode growth medium (NGM) agar plates to agar slides at least twice, first for surgery and then to visualize their axonal regenerative capacity 24 h later^9, 14^. These steps are very time-consuming and hinder the throughput and consistency of laser axotomy experiments in *C. elegans*.

In the last decade, microfluidic platforms have improved the throughput and the repeatability of the experiments in various *C. elegans* studies^18–20^. The assays those microfluidic platforms address include, but are not limited to, lifespan analysis^21, 22^, neuro-developmental studies^23, 24^, phenotyping^25, 26^, behavioral studies^27–29^, electro-physiology^30^, and laser surgery^4, 13, 26, 31^. A few different microfluidic platforms have been developed by our group and others to immobilize the nematode specifically for on-chip laser surgery and interrogation at the cellular and subcellular resolutions^4, 8, 13, 31–33^. For on-chip cellular surgery, Chung *et al*. developed a three-layer microfluidic chip to ablate cell bodies at a rate of 110 worms per hour^31^. To achieve a higher degree of immobilization for subcellular resolution surgery, such as laser axotomy, the most common methodology has been pressurized membranes to exert adequate force for complete immobilization^4, 8, 13, 33^, as fabricated using double-layer soft lithography methods^34, 35^. Membrane based immobilization methods enabled rapid axotomies in a larger number of animals. In a recent study, our group showed that membrane based immobilization microfluidic chips integrated with automation techniques could perform rapid and serial axotomies at a rate of 17 s/worm^13^. Despite the improved immobilization potential and processing rate, multi-layer surgery platforms are difficult to fabricate and operate. Moreover, they require transportation of processed animals out of the chip after the surgery for recovery.

In another attempt of on-chip laser surgery, a single-layer multi-trap microfluidic device has been developed and used to ablate single synapses^32^. This simple chip used a tapered channel geometry to immobilize the animals, however, post-surgery housing and imaging were limited to a couple of hours due to the lack of a separate housing and feeding areas. More importantly, the tapered geometry led the animals to rotate on the anterior-posterior axis plane of their bodies away from the glass interface, as the channel width becomes smaller than its height^36^. This body orientation requires the laser beam to go through the cover glass and a significant portion of the animal body, leading to unsuccessful axotomies and low-contrast imaging of the lateral neurons.

**Figure 1:**
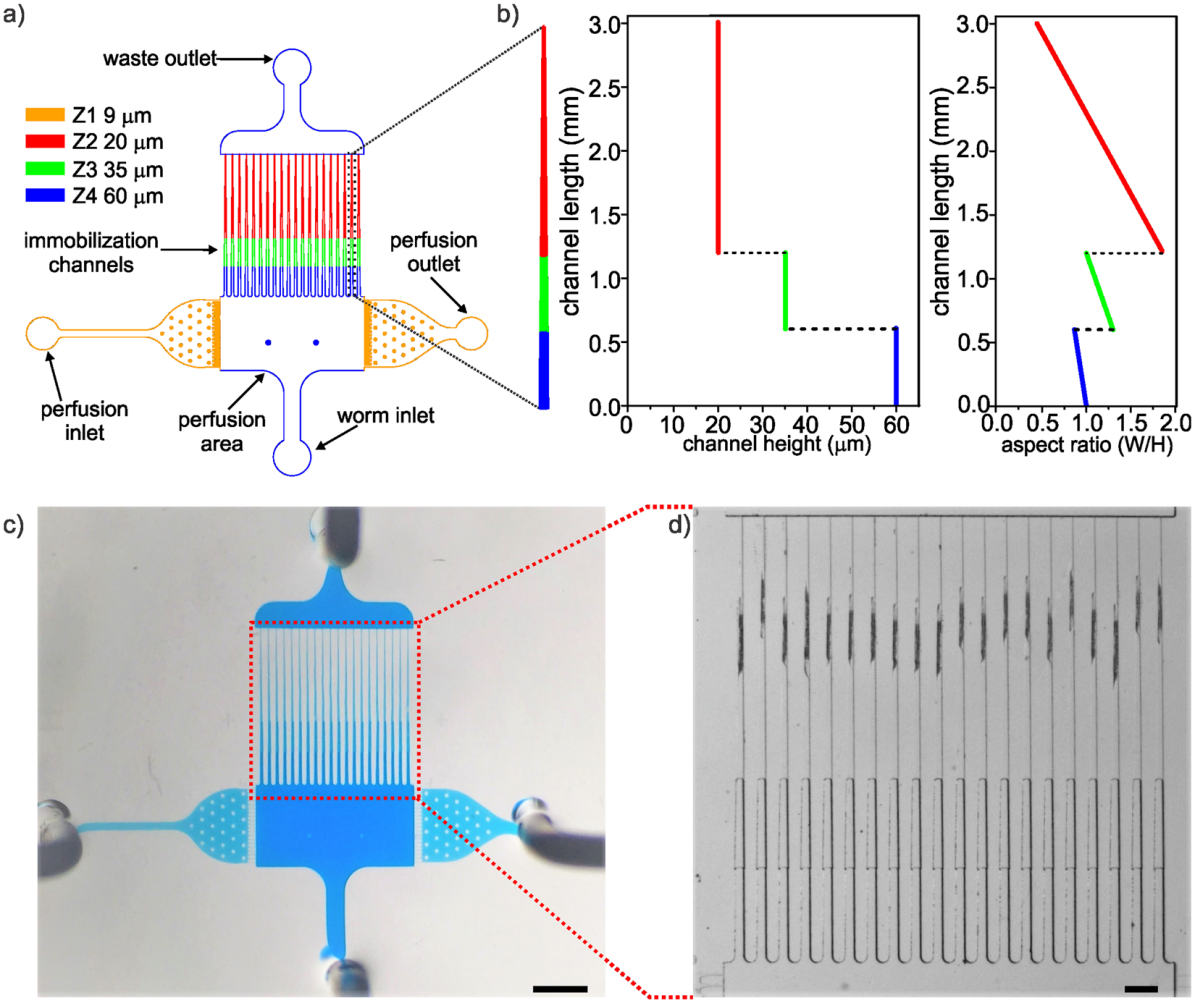
Worm hospital chip. (**a**) A schematic top-view of the worm hospital. The immobilization channels are used to immobilize the nematodes for laser axotomy and post-process imaging. Synchronized animals are loaded into the microfluidic chip through the worm inlet. On-chip feeding during post-surgery is achieved through a pressure gradient between the perfusion inlet and outlet. Different heights are shown in different colors: sieve structures and perfusion inlet and outlets are in yellow (~10 µm), the smallest height in the immobilization channel is in red (20 µm), the second highest channel height is in green (35 µm), and the exit outlet and the perfusion area are in blue (60 µm). (**b**) The channel height (H) and aspect ratio, defined as the ratio of width (W) to channel height (H), along the immobilization channel are represented as a function of channel length. (**c**) Picture of the dye-filled microfluidic chip (scale bar = 1 mm). (**d**) Twenty immobilization channels filled with L4 stage animals with an applied pressure of ~65 kPa (scale bar = 250 µm). All immobilization channels in (**d**) were filled with a single animal.

To overcome these limitations, we developed a new integrated microfluidic device called “worm hospital chip” that allows on-chip axotomy, post-surgery housing, and imaging all on a single device. The chip features 20 immobilization channels, based on a modified tapering channel geometry that we recently proposed and developed^37^, which enables for laser axotomy and subsequent post-surgery imaging, and a perfusion area for housing the worms after laser axotomy. The height of the immobilization channels is reduced in three steps in addition to the continuously tapering channel widths to maintain the aspect ratio around one. This geometry helps the animals to stay in their lateral orientation as they push inside the channels during immobilization. The channels’ dimensions are specifically designed to accommodate both larval stage 4 (L4) animals for on-chip laser axotomy, as well as young adult (YA) stage animals for optical interrogation of neuronal regeneration after 24 h following on-chip housing (Fig. 1a,b). The optimized channel length and reduced flow rates once an animal is trapped ensured immobilization of one single animal inside the immobilization channels. The worm hospital chip is a single-flow layer device and has no active control layers. Its simple design reduces the fabrication and operation complexity of the chip making it more user-friendly than previous multi-flow layer chips, especially for non-expert users. We utilized this chip to test the effect on axonal regeneration of mutations in neurodevelopmental genes of the Wnt/Frizzled family of ligands and receptors. While these molecules are known to be involved in axonal guidance and axonal outgrowth during development^38–46^, only a limited number of studies have been performed to understand their effect after regeneration^9^. Here, we studied in detail how these candidate genes affect axonal repair following a severing injury. The *cwn-2* and *cfz-2* mutations significantly reduced the reconnection rates without any significant effect on the regrowth lengths of the severed axons for both ALM and PLM neurons, whereas the *cwn-1* mutation had a similar effect selectively on ALM neurons.

## Experimental Methods

### Device fabrication

Standard soft-lithography techniques were used to fabricate the single PDMS layer axotomy chip^47^. The resultant photoresist pattern, produced using Mylar masks (FineLine Imaging), was used to create the master mold for all polydimethylsiloxane (PDMS; Sylgard 184, Dow Corning Corp) structures. There were four photolithography steps to create the master mold. To begin, a 4′′ wafer was spin coated with SU-8 2007 photoresist (Microchem Corp.) at 1600 rpm for 33 s to obtain a uniform height of ~10 µm. This layer was exposed to UV light through the first patterned photo-mask to achieve Zone 1 features (Z1, yellow, Fig. 1a) using a mask aligner (MA6/BA6, Suss MicroTec). This layer was then hard baked and developed to remove the unexposed SU8 photoresist. A second layer spun at 4100 rpm atop the Zone 4 feature using SU8-2025 photoresist to obtain a height of ~20 µm. The second layer was exposed to UV light through a photo-mask with the pattern for Zone 2 (Z2, red, Fig. 1a). After the hard bake and development of the second layer, a third layer was spin coated on top of the first two layers using SU8-2025 at 2850 rpm to obtain a uniform height of ~35 µm. This layer was exposed through a unique photo-mask, hard baked, and developed to achieve Zone 3 (Z3, green, Fig. 1a). The fourth layer was spin coated using SU8-2025 photoresist at 1700 rpm to obtain a uniform height of ~60 µm. Similarly, it was exposed through a unique photo-mask, hard baked, and developed to achieve Zone 4 (Z4, blue, Fig. 1a). The resultant four-layer SU8 master mold was treated with tridecafluoro-1,1,2,2-tetrahydrooctyl-1-trichlorosilane vapor (United Chemical Technologies) in a vacuum chamber at −67 kPa and 40 °C to create a hydrophobic layer on the wafer to reduce surface adhesion during the soft-lithography process.

The hydrophobic SU8 master mold was then used to fabricate the PDMS microfluidic chips with soft-lithography techniques. The PDMS (Dow Corning) was mixed at a ratio of 10:1 (base to curing agent), degassed in a vacuum chamber for 20 min to remove bubbles, and slowly poured onto the master to a height of 5 mm. The PDMS layer was cured at 70 °C for 2 h and peeled off from the SU8 mold after cooling. Individual chips were cut out using a razor and fluid interconnect holes were obtained with a 22 gauge punch using a manual punching machine (Accu-Punch MP10-UNV, Syneo). The PDMS chip was then cleaned with tape and irreversibly bonded to a 25 × 50 mm #1.5 cover glass (#2450-1½, Brain Research Laboratories) via oxygen plasma treatment at 100 W for 30 s (PE-50, Plasma Etch Inc.). The bonded device was then cured at 70 °C for 6 h to enhance the glass-PDMS bonding.

### Opto-mechanical setup

The laser axotomy setup incorporates custom optics to deliver femtosecond laser pulses for surgery into a home-built epi-fluorescence microscope as described in our previous works^4, 13^. Briefly, we performed the axotomies and post-surgery imaging using an oil-immersion objective (Plan-Apochromat, 63×, NA = 1.4, Zeiss). For high-resolution imaging of green fluorescent protein (GFP) labeled axons, a mercury arc lamp (XCite 120, EXFO) passing through a FITC filter set (Semrock) provided the excitation light. A three-axis translation stage with three individual actuators (LTA-HS, Newport) operated by a single controller (ESP301-3, Newport), positioned the samples. We used a three-axis piezoelectric actuator (MAX 302, Thorlabs) with a minimal theoretical step size of 25 nm to perform the fine translation. A CCD camera (1392 × 1040 pixels with 6.45 µm pixel size, CoolSnap ES, Photometrics) captured all the images. We carried out all axotomies using a train of 300 pulses with a pulse width of 250 fs and a pulse energy of 7.5 nJ at a center wavelength of 805 nm generated at a repetition rate of 1 kHz (Spitfire, Spectra Physics). The beam energy was measured with an energy meter (PJ10, Ophir) prior to performing all axotomies and adjusted with two pairs of half wave plates and polarizing cube beam-splitters. The high numerical aperture objective tightly focused the laser beam to a spot size of approximately 620 nm based on the theoretical 1/e^2^ diameter of focused Gaussian beams^48^.

### System operation and control

We used a modified version of our previously demonstrated automation software to control the hardware^13^. To control fluid flows in the worm inlet, perfusion inlet, and outlet fluidic channels, we used pressurized external fluid chambers controlled by three-way solenoid valves (The Lee Co., LHDA0521111H) through a manifold (The Lee Co., LFMX0510418). These chambers contained M9 buffer solution (22 mM KH_2_PO_4_, 22 mM Na_2_HPO_4_, 85 mM NaCl, 1 mM MgSO_4_, in dH_2_O). To minimize debris and avoid clogging of the device, all M9 buffer solution was passed through 1.2 µm in-line filters (Acrodisc, Pall Corp.) prior to entering the microfluidic chip. The solenoid valves in fluidic connections were independently actuated with a multichannel amplifier (Automate Scientific). The digital control of the amplifier was carried out by DAQ card (USB6501, National Instruments).

Before each experiment, we primed the device by first introducing M9 buffer solution into the worm inlet and perfusion inlet and outlet. We let M9 buffer solution flow through the exit outlet for ~2 min, which was followed by plugging the exit outlet for ~10 min. This priming procedure helped to eliminate any residual air bubbles within the tubing and the microfluidic chip.

### Laser axotomy and post-surgery imaging

We studied four elements of the axonal regeneration properties of the anterior lateral microtubule cells (ALM) and the posterior lateral microtubule cells (PLM): axonal regrowth (defined by observation of a growth cone stemming from the proximal axon), axonal reconnection (defined as visual contact between proximal and distal axonal fragments), axonal fusion (defined as preservation of the distal fragment after reconnection), and regrowth lengths (measured from the site of surgery to the end of proximal fragment). Depending on the orientation of the immobilized animal, the axotomies were performed on ALM and PLM neurons located on either the left (ALML and PLML) or right (ALMR and PLMR) side of the animal’s body. Axons were injured approximately 60 µm away from the cell body for animals immobilized both on-chip and on-agar using the same opto-mechanical setup. Agar pads were prepared by sandwiching 0.25 mL of melted 4% agar between two microscope slides that were then pulled apart upon cooling to create a flat and uniform surface. We used 10 µL of 10 mM levamisole solution to immobilize ~15 animals for each set of experiments. A coverslip (18 × 18 mm^2^, no. 1.5) was placed on top of the animal just prior to laser axotomy.

For post-surgery nerve regeneration scoring, a z-stack of 14 images (0.8 µm step size) around the severed axon was acquired and saved automatically for each worm. Analysis of the stack of images enabled us to assess the nerve regeneration parameters of the severed axons independent of whether they were located closer to the glass interface or not during the imaging session.

### Nematode maintenance

For mutant studies, *C. elegans* were maintained at 16.5 °C on NGM agar plates seeded with HB101 *E. coli* bacterial culture using standard procedures^49^. We used the following strains for mutant studies: SK4005: *zdIs5* [(*mec-4::gfp*) + *lin-15*(+)], CX6188: *egl-20(n585); zdIs5* [(*mec-4::gfp*) + *lin-15*(+)], CX6219: *mig-1(c1787); zdIs5* [(*mec-4::gfp*) + *lin-15*(+)], CX6429: *cwn-1(ok546);* *zdIs5* [(*mec-4::gfp*) + *lin-15*(+)], CX6787: *cfz-2(ok1201); zdIs5* [(*mec-4::gfp*) + *lin-15*(+)], CX6865: *cwn-2(ok895); zdIs5* [(*mec-4::gfp*) + *lin-15*( + )]. These strains express GFP in the six mechanosensory neurons. Populations of age-synchronized animals were prepared by collecting and isolating embryos following hypochlorite treatment. Gravid adults were lysed with a small volume of a 2:1 mixture of sodium hypochlorite and 4 M sodium hydroxide, and the collected eggs were suspended in M9 buffer overnight on a rotator to aerate. The embryos were hatched overnight and were arrested in the larval stage 1 (L1) until the food was reintroduced. The L1 stage worms were then placed on HB101-seeded agar plates and allowed to grow for 48-50 hours until the worms reached the early to the middle L4 stage.

### On-chip immobilization

Laser axotomy and post-surgery imaging of *C. elegans* need a high-degree immobilization. The new single layer axotomy chip provides immobilization of animals by geometrically restricting them inside tapering micro-channels. Individual animals are pushed inside these channels until the walls of the shortest channel section (Z2 in red, Fig. 1a,b) prevent any sinusoidal body movement such as crawling or swimming^22^. Preventing the sinusoidal body movement provides enough immobilization for axotomy on ALM neurons, located near the middle part of the worm body. However, PLM neurons are located near the tail, having space to move (Supplementary Movie 1). To eliminate the motility of the tail of trapped animals, we used a low concentration of levamisole (0.625 mM levamisole) to perform axotomy on the PLM neurons (Supplementary Movie 2). We prepared the animal suspension in M9 buffer solution with 0.625 mM levamisole as follows. First, 1 ml of M9 solution was pipetted onto animals growing on an agar pad. Second, 0.5 ml of this M9 buffer solution suspended with worms was drawn into a syringe tube containing 0.5 ml of 1.25 mM levamisole. As a result, we obtained ~50–75 animals suspended in 1 ml of 0.625 mM levamisole solution. In addition, we used 0.625 mM levamisole in an external fluid reservoir to provide buffer flow through the worm inlet during on-chip PLM axotomy and imaging.

### Data availability

The data that support the findings of this study are available from the corresponding author on request.

## Results and Discussion

### Worm hospital microfluidic chip design

The chip constitutes two main compartments, enabling complete nerve regeneration studies inside a single device without the need for transferring them between the axotomy, recovery, and follow-up imaging procedures. The first compartment includes a set of 20 parallel immobilization channels for axotomy and imaging of the nematodes, and the second compartment includes a perfusion area for their housing and feeding after the axotomy and during post-surgery recovery (Fig. 1a).

The immobilization channels were carefully designed to accommodate three experimental requirements. (1) Immobilize the animals with a desired lateral orientation to ensure their axons are in close proximity of the glass interface for optimal optical access, (2) achieve a high degree of immobilization of L4 as well as YA stage animals, which have substantially different dimensions, and (3) trap and immobilize only a single animal in each channel. To fulfill these conditions, we adapted a modified tapered geometry design that we had recently developed, where we could trap and immobilize thousands of animals in a large scale microfluidic chip with a pressure-driven flow^37^. While the early design of tapered channel geometry ensured the high-degree immobilization^22^, we found that the arbitrary orientation of the worms was unfavorable for axotomy and post-surgical imaging^36^. The monotonically decreasing aspect ratio of the channels^21, 22, 32^ caused the animals to rotate their lateral axis plane away from the cover glass interface^36^. To get the best optical access to the lateral neurons (ALM or PLM) and avoid rotation of the animals during immobilization, we reduced the height of the channels in addition to their width, keeping the aspect ratio around one^37^ (Fig. 1b).

The perfusion area incorporates the fourth layer in the perfusion inlet and outlet (shown in yellow, Fig. 1a) to successfully house the animals over a 24 h period for follow-up imaging of the regenerating axons. This layer consists of 20 sieve channels on the sides of the perfusion area through which it connects the perfusion inlet to the outlet. The sieve structures on each arm have an opening of 8 μm × 10 μm (width × height), which are small enough to block animals from passing through them but allow the liquid culture medium to perfuse food through the feeding chamber.

We designed the perfusion arms asymmetrically to obtain different hydraulic resistance values. The low resistance arm (perfusion outlet) was essential to remove animals carefully from the immobilization channels after surgery without using high pressure. The high resistance arm (perfusion inlet) was necessary to reduce the food perfusion flow rate to approximately 1 nl/s, which was determined empirically. The food perfuses through a positive pressure gradient between the inlet and outlet perfusion arms. A 5-mL fluid reservoir with a bacterial food suspension provides the pressure gradient to the perfusion inlet. The reservoir fluid line is 4 cm above the opening of the tubing that is connected to the perfusion outlet. The slow perfusion rate allows the animals to swim freely and feed without excessive stress or having their bodies pushed up against the sieve structures during the post-surgery incubation period. The circular pillars in the perfusion area and side perfusion arms provide structural stability to avoid the collapse of the large PDMS chambers during fabrication or operation of the device.

We fabricated a single SU-8 master mold incorporating features at four different heights. This single layer PDMS microfluidic chip offers simple fabrication and lower device-to-device variation than multilayer PDMS microfluidic chips (Fig. 1c,d). Worm inlet and exit channels were fabricated to a thickness of 60 μm (shown in blue; Fig. 1a) to reduce physical stress on the animals inside the chip during the loading process and post-surgery incubation, and to avoid any undesired clogging in the exit area. The narrowest channel section (Z2 in red, Fig. 1a) was designed with the longest channel length of 1.8 mm (Fig. 1b). The relatively long channel ensured the trapping and immobilization of animals at different developmental stages, ranging from young L4 (for initial trapping and axotomy) to YA (for re-trapping and imaging subsequent axon regeneration).

### Microfluidic flow sequence

**Figure 2:**
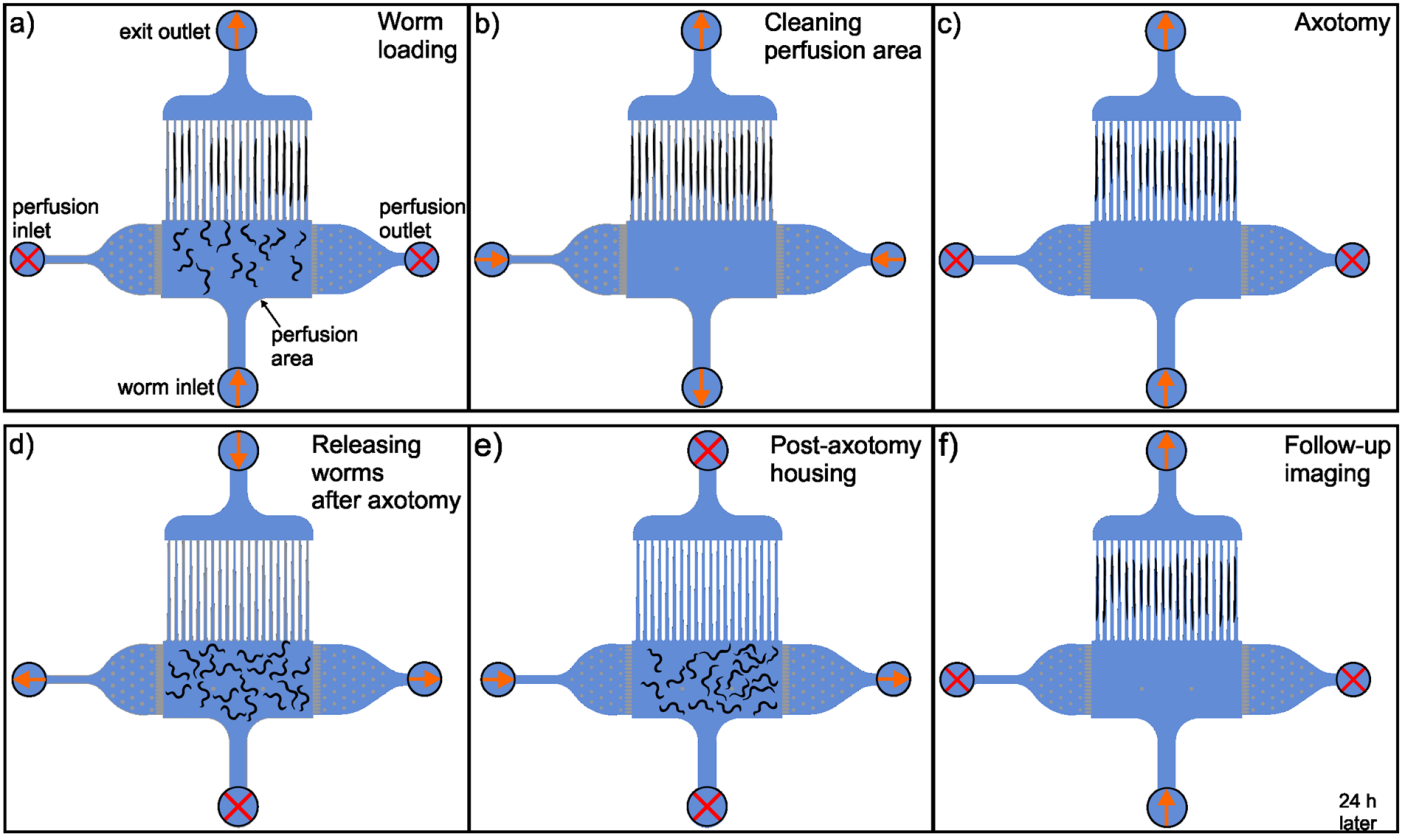
On-chip sequence of valve actuations and flow progressions. (**a**) The initial step involves loading of animals from a single population in the perfusion area, and then pushing them into the immobilization chambers using a syringe. Blocking the perfusion connections during loading and immobilization helps to have a uni-directional flow between the worm inlet and the exit outlet. (**b**) After filling most of the immobilization chambers, the remaining worms are removed out of the microfluidic chip by opening the worm inlet and applying pressure from the perfusion connections. (**c**) Following the cleaning of the perfusion area, laser axotomy is performed on each immobilized worm. Trapping and immobilization are sustained with the pressure gradient between the worm inlet and the exit outlet throughout the process. (**d**) The axotomized worms are then released back into the perfusion area for on-chip feeding and housing by creating a pressure gradient between the exit outlet and the perfusion connections. (**e**) During post-axotomy housing, a slow perfusion between the perfusion inlet and outlet feeds the animals for the entire duration of recovery. (**f**) For follow-up imaging of the nerve regeneration phenotype, we bring back the chip to the microscope and push the animals into the immobilization channels.

The sequence of valve actuations and flow progressions for each step are shown in Fig. 2. A population of ~50–70 animals suspended in M9 buffer solution is loaded through the main inlet into the individual immobilization channels by blocking the flow at the perfusion connections (Fig. 2a). This step takes approximately 3 min. After filling most channels with a single animal, extra ones in the perfusion area are pushed out of the chip by opening the worm inlet to atmosphere and pressurizing the perfusion inlet and outlet with a pressure of ~100 kPa (Fig. 2b). Before axotomies, the trapped animals are pushed further inside the channels and immobilized by pressurizing the main inlet at ~55 kPa and closing the perfusion connections (Fig. 2c). The pressure gradient between the worm inlet and waste outlet provides the required level of immobilization to perform precise laser axotomy on each immobilized animal (Fig. 2c). Upon completion of axotomies, the animals are returned into the perfusion area for post-axotomy recovery by pressurizing the waste outlet and letting the flow go through the sieve structures. The axotomized animals are housed and fed on-chip (Fig. 2d) via a pressure gradient between perfusion inlet and outlet while the main inlet and the waste outlet connections are blocked for 24 h (Fig. 2e). To provide a constant supply of food (bacteria) in S medium, the perfusion inlet is connected to a 5 ml syringe, filled with bacteria in S medium, and the perfusion outlet is connected to atmospheric pressure^21^. After 24 h of on-chip housing and feeding, the animals are pushed once more into the immobilization channels by pressurizing the main inlet for high-resolution post-surgery recovery imaging of axon regeneration (Fig. 2f).

### Trapping efficiency and orientation characterization

The overall success of this on-chip process is defined by the efficiency of initial trapping of animals prior to axotomy and the efficiency of re-trapping housed worms for imaging 24 h later. For achieving high efficiencies for both trapping and re-trapping, we iterated the chip design several times. For example, we optimized the length of the immobilization channels to overcome the problem of multiple animals filling the same channel, and also incorporated a fourth PDMS layer to shorten the height of the sieve structures to keep the axotomized worms inside the perfusion area while providing an optimum flow rate for feeding (Fig. S1). These design iterations led us to a single layer microfluidic device (Fig. 1) achieving the desired performance for trapping both small L4 (30 μm in diameter, 600 μm in length) and larger YA stage animals (40 μm in diameter, 800 μm in length), while allowing one single animal in each immobilization channel.

**Figure 3:**
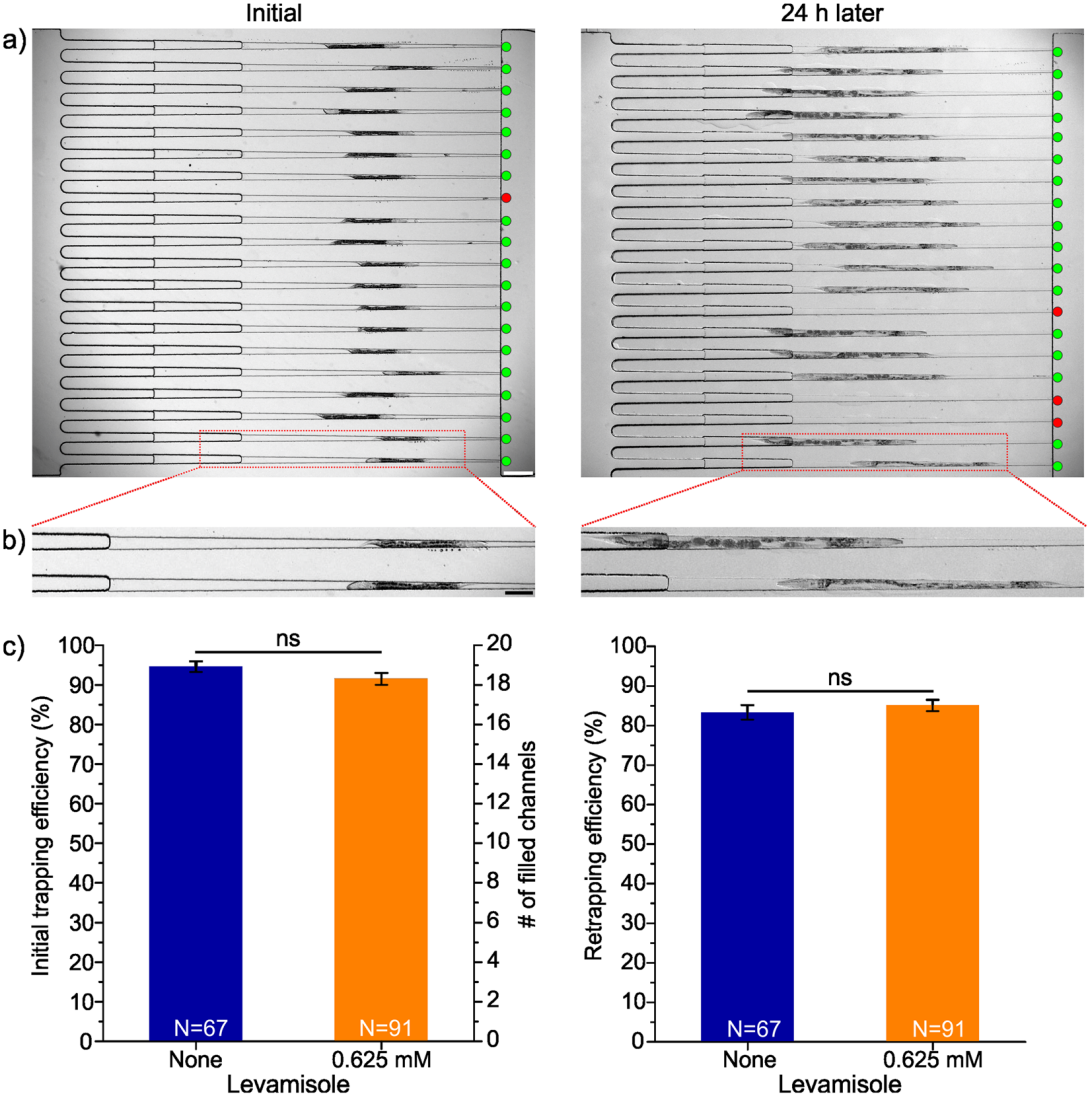
Trapping and re-trapping efficiencies. (**a**) Top-views of parallel immobilization channels with immobilized young L4 (left) and young adult (YA) stage animals after 24 h on-chip housing (right). In this specific case, out of 20 single immobilization channels, 19 contained a single immobilized animal (marked with green circles) and only one of the channels was empty (marked with red circles). Out of 19 initially processed animals, 17 were successfully re-trapped (scale bar = 200 µm). (**b**) The zoomed-in images show two adjacent channels each containing a single immobilized animal with different head-and-tail orientations. To show the difference in the size of animals of different stages we kept the zoomed-in images the same size (scale bar = 100 µm). (**c**) The initial trapping efficiencies (left) with and without anesthetics show no statistically significant (ns) difference. On-chip re-trapping efficiencies (right) with and without anesthetics show a recovery and re-trapping at an average of 84% of the axotomized worms independently of the use of anesthetics. Error bars show standard error of mean (SEM), and the number in each bar indicates the number of performed experimental sets – each set representing individual chip run. We used a two-tailed *t*-test for statistics (ns – not significant).

In general, we observed a very high initial-trapping efficiency of the L4 stage animals and achieved desired lateral orientation during initial immobilization, which was necessary to perform the highest precision axotomy (Fig. 3a). The trapping efficiency slightly reduced for YA animals after 24 h recovery as their volume nearly doubled due to 25% increase in both their diameter and length (Fig. 3b).

Figure 3c shows the quantitative results of the initial trapping and re-trapping efficiencies. Since we used a small amount of levamisole for axotomies of PLM neurons, we characterized the trapping efficiencies for both immobilization conditions, without levamisole and with 0.625 mM levamisole over 158 different sets of experiments. On average, each chip was used for 4 experiments until the cleaning of small sieve structures was insufficient to obtain a fully functioning chip.

The initial trapping efficiency was 94.6 ± 1.1% (N = 67 experiments) for the no-levamisole condition, and 91.9 ± 1.0% (N = 91 experiments) for the 0.625 mM levamisole condition (Fig. 3c). The main reasons for unsuccessful trapping were (i) clogging of the immobilization channels during animal loading, and (ii) trapping of more than one animal in a single channel. Clogging was mainly caused by the introduction of agar particulates, whereas multi-animal trapping in a single channel was due to varying size of animals within the population.

The re-trapping efficiencies were slightly lower than the initial trapping ones, ranging from 83.3 ± 2.0% (N = 67 experiments) to 85.3 ± 1.5% (N = 91 experiments) for the no-levamisole and 0.625 mM levamisole conditions, respectively. The main reasons for unsuccessful re-trapping were (i) the difficulty of re-trapping YA animals that were substantially larger than the L4 stage animals, and (ii) the loss of animals during the 24 h recovery time. Relatively low perfusion rates were needed to keep the animals healthy in the housing chamber but it resulted in loss of animals as they could freely move up the tubing connected to the main inlet.

There was no statistically significant difference between two trapping efficiencies of both groups. The overall efficiencies of the entire on-chip processes were 78.8% and 78.4% for no levamisole and with 0.625 mM levamisole, respectively. In other words, on average we could study nerve regeneration in 16 animals on chip with 20 immobilization channels. All trapped animals were successfully oriented on their lateral side as desired, and with an equal probability for their head/tail to be towards the exit outlet (Fig. S2).

### Effect of on-chip immobilization and housing on worm viability

We carried out a viability test to assess the effect of on-chip immobilization and 24 h on-chip housing on worm survival, with and without the use of levamisole, and compared them to a control group of *zdIs5 (mec-4::GFP*, expressing GFP in six mechanosensory neurons) animals that were not subjected to any on-chip processing. We subjected both groups to an initial immobilization for 20 min with a pressure of 55 kPa, on-chip housing for 24 h, and re-immobilization for 20 min again with a pressure of 55 kPa. After re-immobilization, we flushed both trial groups out of the chip and collected them on NGM plates. We checked the viability of the worms in each population every 24 h and transferred them to new NGM plates. The average lifespans of the immobilized groups were 19.8 ± 3.7 and 19.1 ± 5.0 days for on-chip immobilization with no levamisole and 0.625 mM levamisole, respectively, whereas the average lifespan of the control group was 19.2 ± 3.8 days. We used the log-rank test to determine the difference between the viabilities of each group. We found no statistically significant differences (*p* > 0.90) in the outcome for both groups (Fig. S3).

### Effect of on-chip immobilization and housing on axonal regeneration rates

**Figure 4:**
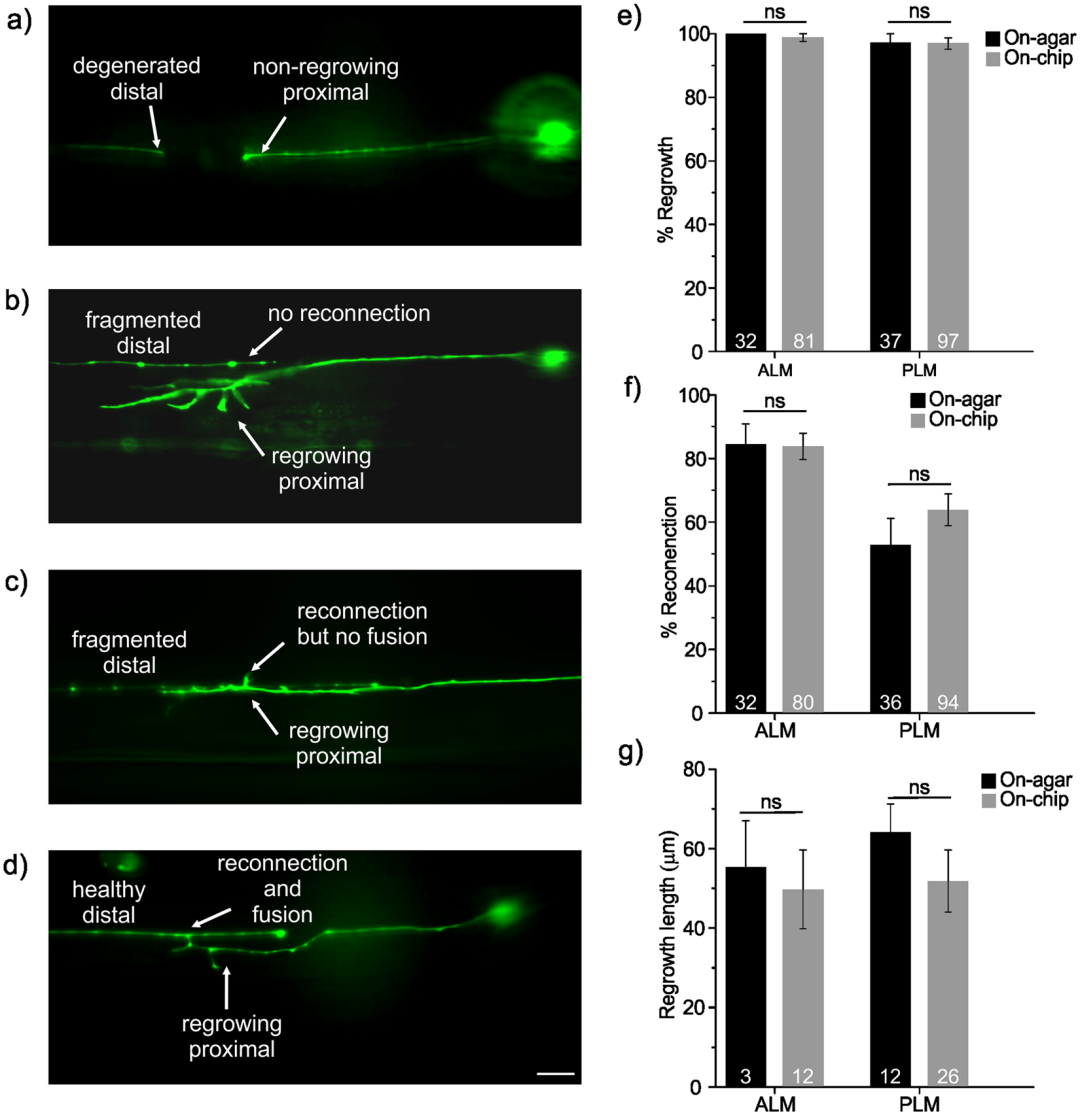
Effect of on-chip immobilization and housing on axonal regeneration properties. (**a**) A fluorescence image of a non-regrowing PLM neuron of an *egl-20* mutant animal, showing a lack of a growth cone formation and regrowth beyond the injury site. (**b**) A fluorescence image of a regrowing ALM neuron of a *cfz-2* mutant animal without any reconnection and fusion, as evident by the proximal end regrowing in a substantially different focal plane than the distal end, and the visible fragmented beading in the distal part. (**c**) A fluorescence image of a regrowing PLM neuron of a *cwn-2* mutant animal, which seems to be in the close proximity of distal part, namely reconnected, but not fused to the distal part. (**d**) A fluorescence image of a regrowing PLM neuron of a *zdIs5* animal, showing a successful connection and fusion as determined by the healthy distal part. The fusion point of distal and proximal ends is indicated by the arrow. (**e**) Axonal regrowth rates, (**f**) reconnection rates, and (**g**) regrowth lengths of the ALM and PLM neurons of *zdIs5* transgenic animals studied on-chip and on-agar conditions. The error bars show standard error of proportion, and the number in each bar indicates the number of animals. For statistics, we used a two-tailed *t*-test (ns, not significant). Scale bar is 10 µm.

Before starting the axonal regeneration studies on the new chip, we analyzed whether on-chip axotomy by itself affected this process by comparing the regeneration parameters to those axotomies performed on-agar as a control (Fig. 4). We determined four regeneration parameters using the following criteria: (1) axonal regrowth, as the appearance of a growth cone (Fig. 4a,b), (2) axonal reconnection, as proximity of re-growing proximal axon with the distal axon (Fig. 4b,c), (3) axonal fusion, as the lack of beading or fragmentation in the distal axon (degeneration)^10, 14^ (Fig. 4d), and (4) axonal regrowth lengths, as the length of those regrowing axons that did not fuse. The regrowth length measurement included the regrowth length from the injury site as well as the lengths of filopodia.

To determine the effect of the worm hospital on axonal regeneration, we specifically used the transgenic strain *zdIs5*, expressing GFP in six touch receptor neurons, as our control. The results presented in Fig. 4e–g show no statistically significant differences for any of these parameters between axotomies performed on-agar using anesthetics and those carried out on-chip for both ALM and PLM neurons.

### Regeneration properties of axonal growth and guidance, and neuronal polarity genes

The connectivity and formation of the nervous system are regulated by phylogenetically conserved molecules^38, 39, 50^. The Wnt ligands and their canonical Frizzled receptors have been shown to regulate in different species several developmental processes, including cell migration, neuronal polarity, axonal guidance, and axonal branching^38–43, 50–53^. There are five Wnt ligands (LIN-44, EGL-20, CWN-1, CWN-2, and MOM-2) and four Frizzled receptors (MIG-1, CFZ-2, MOM-5, and LIN-17) present in the *C. elegans* genome, and one or more mutant alleles are available for each of them^53^. We hypothesized that some of the molecules of Wnt/Frizzled family might also be involved in the axonal regeneration process. Only a few of these developmental genes were screened for their effect on the neuronal regrowth capability of PLM neurons^9^, and we decided to determine their role in axonal regeneration, reconnection and fusion in two different mechanosensory neurons, ALM and PLM.

**Figure 5:**
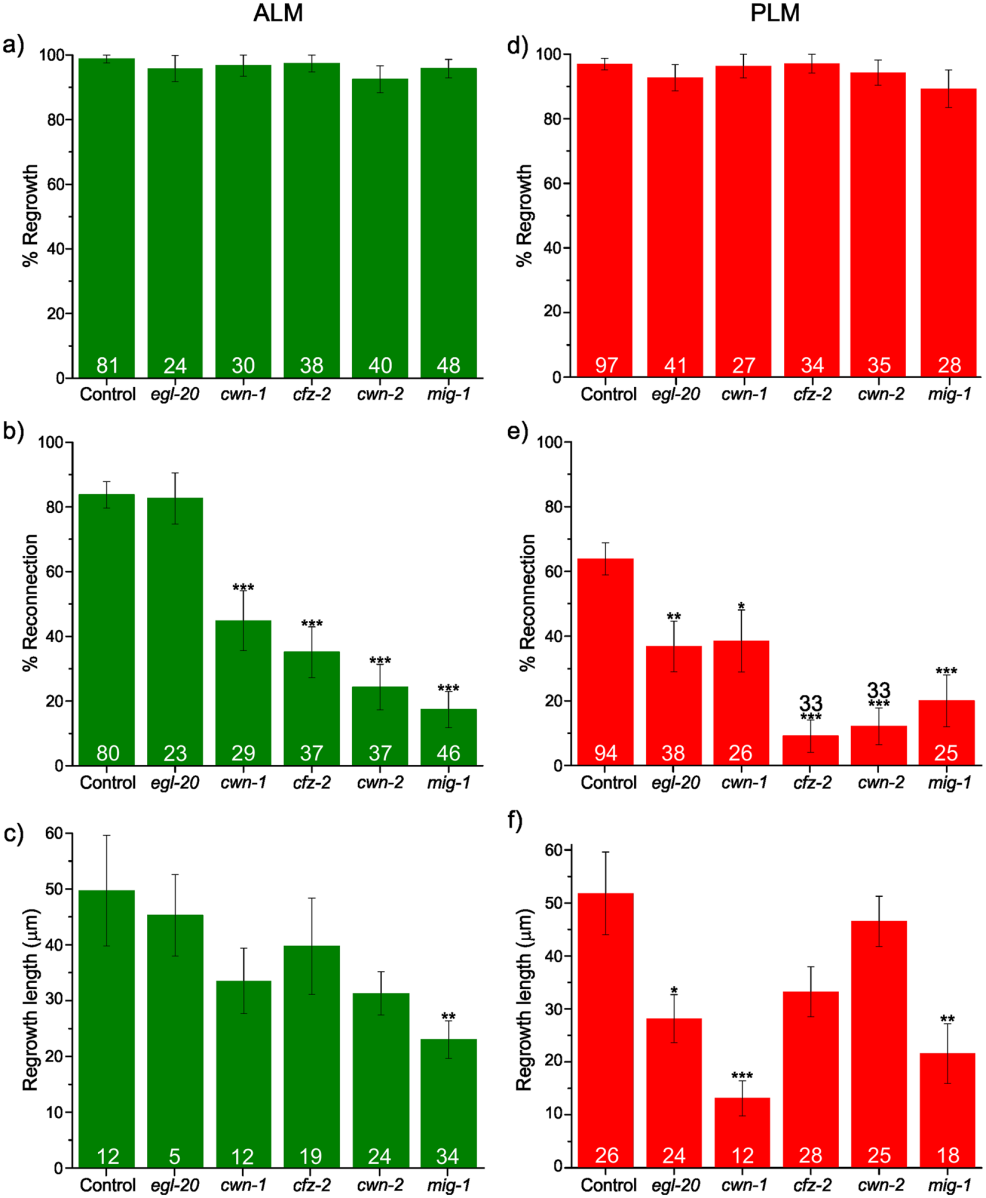
On-chip nerve regeneration results for the selected genes of the WNT/Frizzled family. Quantification of the successful regrowth rates is shown in (**a**) and (**d**) for ALM and PLM neurons, respectively. Quantification of the successful reconnection rates of the regrowing axons is shown in (**b**) and (**e**) for ALM and PLM neurons, respectively. Regrowth lengths of the regrowing axons that did not fuse are shown in (**c**) and (**f**) for ALM and PLM neurons, respectively. The numbers adjacent to each bar indicate the total number of animals processed. The error bars in (**a**), (**b**), (**d**), and (**e**) show standard error of proportion and the number in each bar indicates the number of animals. The error bars in (**c**) and (**f**) show standard error of mean. We used one-way ANOVA with Dunnett’s correction for multiple comparisons for (**a**), (**b**), (**d**), and (**e**). We used Mann-Whitney test for (**c**) and (**f**). The *p* values for ANOVA or Mann-Whitney test are: 0.01 < *p* < 0.05 (*), 0.001 < *p* < 0.01 (**), and *p* < 0.001 (***).

We studied single mutant strains of five genes in the *zdIs5* background: *cwn-1, cwn-2, egl-20, mig-1, cfz-2* using the worm hospital chip following the protocol described above (Fig. 5). First, we analyzed whether these genes affected the regrowth rate defined by the formation of a growth cone after severing the axons. For every mutant tested, we observed no significant effect in regrowth percentages for both ALM and PLM 24 h after the laser axotomy (Fig. 5a,d, Supplementary Tables 3 and 4). Next, we analyzed whether the neurons that displayed a successful regrowth also underwent a visible reconnection with their separated distal fragment 24 h after the laser axotomy. The reconnection could only be visualized within the available optical resolutions of 350–400 nm, where the proximal and distal ends looked visually in close proximity to each other. We found that in both ALM and PLM neurons the percentage of reconnection was significantly decreased in every mutant tested except *egl-20* for the ALM neurons (Fig. 5b,e, Supplementary Tables 5 and 6). We then assessed whether the neurons that displayed a successful reconnection to their distal part also showed a successful axonal fusion. Most reconnected axons in both ALM and PLM neurons showed healthy distal ends, indicating that none of the five genes significantly reduced the axonal fusion rates (Fig. S5, Supplementary Tables 7 and 8). The mutations significantly reduced the reconnection percentage in most genes, led to a low number of animals to be studied for the axonal fusion phenotype. Thus, a larger scale axotomy assay may be pursued to understand the effects of the studied genes on the axonal fusion phenotype.

In summary, our results suggest that the capacity of sprouting a growth cone, as well as fusing with their own separated distal process, are not altered by mutations in any of these individual genes. Second, similarly to what happens during development, these molecules might be involved in regulating the correct guidance of the regenerating axons, except for *EGL-20* in the ALM neurons.

Lastly, we measured the regrowth lengths, focusing on those axons that regrew but did not undergo a successful fusion. In both ALM and PLM neurons, *mig-1* mutation significantly reduced the regrowth lengths of the severed axons (Fig. 5c,f, Supplementary Tables 9 and 10). In addition, mutations in *cwn-1* and *egl-20* significantly reduced the regrowth lengths in PLM neurons. Previous studies also showed a significant reduction in regrowth length of severed axons in *egl-20* ^9^, but not in *cwn-1*. Interestingly, *cwn-1* mutant animals did not reduce the regrowth length in ALM neurons despite the significant reduction in the reconnection rates. A similar phenotype was observed with the *cwn-2* and *cfz-2* mutant animals, where a significant reduction in the reconnection rates did not reduce significantly the regrowth length of the severed axons. These results suggest that the *cwn-2* and *cfz-2* mutations in both neurons, along with the *cwn-1* mutation in ALM neurons reduce the reconnection rate by possibly affecting the guidance of the severed axons without inhibiting the capabilities of regrowth length (Supplementary Tables 11 and 12).

## Conclusions

We designed, implemented, and characterized a worm hospital chip with multiple arrays for longitudinal studies of nerve regenera﻿tion in *C. elegans*. The microfluidic platform allows on-chip axotomy, post-axotomy housing, and post-surgical recovery imaging of nerve regeneration after 24 h, all inside the same chip. The trapping efficiency for axotomy is at least 91% with and without using levamisole as anesthetics, while orienting all of them in their lateral side for best imaging and axotomy conditions. After 24 h of on-chip housing, the re-trapping efficiency of axotomized animals exceeds 83%.

Using the worm hospital chip, we studied the effect of several developmental genes of the Wnt/Frizzled family for their role on the  axonal regeneration capability of the touch receptor neurons (ALM and PLM). We observed that most, but not all, genes in Wnt/Frizzled family involved in developmental process are also essential for the axonal regeneration and successful axonal reconnection after an injury for both ALM and PLM neurons. Specifically, the mutations *cwn-1, cwn-2, cfz-2*, and *mig-1* significantly decreased the reconnection rate for both ALM and PLM neurons, whereas *egl-20* mutation significantly reduced reconnection rate only in PLM neurons. Additionally, we observed that the *cwn-2* and *cfz-2* mutant animals showed a significant reduction in the reconnection rates without any significant reduction in the regrowth lengths of the severed axons for both neurons. A similar effect was observed for *cwn-1* mutant animals in ALM neurons only.

The new worm hospital chip offers an easy-to-use microfluidic platform especially for non-expert users. Its versatility enables nerve regeneration studies of any other neurons such as AVM or PVM neurons, and the neuronal processes located along the ventral cord. The unique capability of integrating the axotomy, housing, and post-recovery surgery imaging in a single chip is promising for future high-throughput studies.

### Statistical analysis

Error of proportions was used to assess variation across a single population and two-way comparison was performed using the *t*-test. For more than two groups, one-way ANOVA with Dunnett’s *post-hoc *test for multiple comparisons was used. For those data sets that did not follow a normal distribution, we used the Mann-Whitney test (Figs. 4g and 5c,f).

## Electronic supplementary material

Supplementary Information

Supplementary Movie 2

Supplementary Movie 1

## References

[1] Silver J, Miller JH (2004). Regeneration beyond the glial scar. Nature Reviews Neuroscience.

[2] Yiu G, He Z (2006). Glial inhibition of CNS axon regeneration. Nature Reviews Neuroscience.

[3] Yanik MF (2004). Neurosurgery: functional regeneration after laser axotomy. Nature.

[4] Guo SX (2008). Femtosecond laser nanoaxotomy lab-on-a-chip for *in vivo* nerve regeneration studies. Nature Methods.

[5] Yan D, Wu Z, Chisholm AD, Jin Y (2009). The DLK-1 Kinase Promotes mRNA Stability and Local Translation in *C. elegans* Synapses and Axon Regeneration. Cell.

[6] Hammarlund M, Nix P, Hauth L, Jorgensen EM, Bastiani M (2009). Axon regeneration requires a conserved MAP kinase pathway. Science.

[7] Ghosh-Roy A, Wu Z, Goncharov A, Jin Y, Chisholm AD (2010). Calcium and cyclic AMP promote axonal regeneration in *Caenorhabditis elegans* and require DLK-1 kinase. The Journal of Neuroscience.

[8] Samara C (2010). Large-scale *in vivo* femtosecond laser neurosurgery screen reveals small-molecule enhancer of regeneration. Proceedings of the National Academy of Sciences.

[9] Chen L (2011). Axon Regeneration Pathways Identified by Systematic Genetic Screening in *C. elegans*. Neuron.

[10] Neumann B, Nguyen KC, Hall DH, Ben‐Yakar A, Hilliard MA (2011). Axonal regeneration proceeds through specific axonal fusion in transected *C. elegans* neurons. Developmental Dynamics.

[11] El Bejjani R, Hammarlund M (2012). Notch signaling inhibits axon regeneration. Neuron.

[12] Nix P (2014). Axon regeneration genes identified by RNAi screening in *C. elegans*. The Journal of Neuroscience.

[13] Gokce SK (2014). A fully automated microfluidic femtosecond laser axotomy platform for nerve regeneration studies in *C. elegans*. PLoS One.

[14] Neumann B (2015). EFF-1-mediated regenerative axonal fusion requires components of the apoptotic pathway. Nature.

[15] Lewis J, Wu C-H, Levine J, Berg H (1980). Levamisole-resitant mutants of the nematode *Caenorhabditis elegans* appear to lack pharmacological acetylcholine receptors. Neuroscience.

[16] Byrne, A. B., Edwards, T. J. & Hammarlund, M. *In vivo* laser axotomy in *C. elegans*. *Journal of visualized experiments: JoVE* (2011). 10.3791/2707 PMC3168200 21633331

[17] Kim E, Sun L, Gabel CV, Fang-Yen C (2013). Long-term imaging of *Caenorhabditis elegans* using nanoparticle-mediated immobilization. PLoS One.

[18] Ben-Yakar A, Chronis N, Lu H (2009). Microfluidics for the analysis of behavior, nerve regeneration, and neural cell biology in *C. elegans*. Current Opinion in Neurobiology.

[19] Hulme SE, Whitesides GM (2011). Chemistry and the worm: *Caenorhabditis elegans* as a platform for integrating chemical and biological research. Angewandte Chemie International Edition.

[20] Bakhtina NA, Korvink JG (2014). Microfluidic laboratories for *C. elegans* enhance fundamental studies in biology. RSC Advances.

[21] Hulme SE (2010). Lifespan-on-a-chip: microfluidic chambers for performing lifelong observation of *C. elegans*. Lab on a Chip.

[22] Hulme SE, Shevkoplyas SS, Apfeld J, Fontana W, Whitesides GM (2007). A microfabricated array of clamps for immobilizing and imaging *C. elegans*. Lab on a Chip.

[23] Mondal S, Ahlawat S, Rau K, Venkataraman V, Koushika SP (2011). Imaging *in vivo* neuronal transport in genetic model organisms using microfluidic devices. Traffic.

[24] de Carlos Cáceres, I., Valmas, N., Hilliard, M. A. & Lu, H. Laterally orienting *C. elegans* using geometry at microscale for high-throughput visual screens in neurodegeneration and neuronal development studies. *PLoS One* **7** (2012). 10.1371/journal.pone.0035037 PMC3335040 22536350

[25] Chung K, Crane MM, Lu H (2008). Automated on-chip rapid microscopy, phenotyping and sorting of *C. elegans*. Nature Methods.

[26] Crane MM, Chung K, Lu H (2009). Computer-enhanced high-throughput genetic screens of *C. elegans* in a microfluidic system. Lab on a Chip.

[27] Chronis N, Zimmer M, Bargmann CI (2007). Microfluidics for *in vivo* imaging of neuronal and behavioral activity in *Caenorhabditis elegans*. Nature Methods.

[28] Lockery SR (2008). Artificial dirt: microfluidic substrates for nematode neurobiology and behavior. Journal of neurophysiology.

[29] Vidal-Gadea, A. *et al*. Magnetosensitive neurons mediate geomagnetic orientation in *Caenorhabditis elegans*. *eLife*, e07493 (2015). 10.7554/eLife.07493 PMC4525075 26083711

[30] Lockery SR (2012). A microfluidic device for whole-animal drug screening using electrophysiological measures in the nematode *C. elegans*. Lab on a Chip.

[31] Chung K, Lu H (2009). Automated high-throughput cell microsurgery on-chip. Lab on a Chip.

[32] Allen PB (2008). Single-synapse ablation and long-term imaging in live *C. elegans*. Journal of Neuroscience Methods.

[33] Zeng F, Rohde CB, Yanik MF (2008). Sub-cellular precision on-chip small-animal immobilization, multi-photon imaging and femtosecond-laser manipulation. Lab on a Chip.

[34] Unger MA, Chou H-P, Thorsen T, Scherer A, Quake SR (2000). Monolithic microfabricated valves and pumps by multilayer soft lithography. Science.

[35] Thorsen T, Maerkl SJ, Quake SR (2002). Microfluidic large-scale integration. Science.

[36] Ghorashian, N. Parallelized microfluidic devices for high-throughput nerve regeneration studies in Caenorhabditis elegans Master of Science Thesis thesis, UT Austin, (2010).

[37] Mondal, S. *et al*. Large-scale microfluidics providing high-resolution and high-throughput screening of *Caenorhabditis elegans* poly-glutamine aggregation model. *Nature Communications* **7** (2016). 10.1038/ncomms13023 PMC5062571 27725672

[38] Serafini T (1994). The netrins define a family of axon outgrowth-promoting proteins homologous to *C. elegans* UNC-6. Cell.

[39] Tessier-Lavigne M, Goodman CS (1996). The molecular biology of axon guidance. Science.

[40] Maloof JN, Whangbo J, Harris JM, Jongeward GD, Kenyon C (1999). A Wnt signaling pathway controls hox gene expression and neuroblast migration in *C. elegans*. Development.

[41] Whangbo J, Kenyon C (1999). A Wnt signaling system that specifies two patterns of cell migration in *C. elegans*. Molecular cell.

[42] Hilliard MA, Bargmann CI (2006). Wnt Signals and Frizzled Activity Orient Anterior-Posterior Axon Outgrowth in *C. elegans*. Developmental cell.

[43] Pan C-L (2006). Multiple Wnts and Frizzled Receptors Regulate Anteriorly Directed Cell and Growth Cone Migrations in *Caenorhabditis elegans*. Developmental cell.

[44] Tabara H, Grishok A, Mello CC (1998). RNAi in *C. elegans*: soaking in the genome sequence. Science.

[45] Winston WM, Molodowitch C, Hunter CP (2002). Systemic RNAi in *C. elegans* requires the putative transmembrane protein SID-1. Science.

[46] Lyuksyutova AI (2003). Anterior-posterior guidance of commissural axons by Wnt-frizzled signaling. Science.

[47] Xia Y, Whitesides GM (1998). Soft lithography. Annual review of materials science.

[48] Urey H (2004). Spot size, depth-of-focus, and diffraction ring intensity formulas for truncated Gaussian beams. Applied optics.

[49] Brenner S (1974). The genetics of *Caenorhabditis elegans*. Genetics.

[50] Dickson BJ (2002). Molecular mechanisms of axon guidance. Science.

[51] Whangbo J, Harris J, Kenyon C (2000). Multiple levels of regulation specify the polarity of an asymmetric cell division in *C. elegans*. Development.

[52] Ch’ng Q (2003). Identification of genes that regulate a left-right asymmetric neuronal migration in *Caenorhabditis elegans*. Genetics.

[53] Korswagen HC (2002). Canonical and non‐canonical Wnt signaling pathways in *Caenorhabditis elegans*: Variations on a common signaling theme. Bioessays.

