# Supplementary material for: A multi-trap microfluidic chip enabling longitudinal studies of nerve regeneration in Caenorhabditis elegans
Source: Sci Rep. 2017 Aug 29;7:9837. doi: 10.1038/s41598-017-10302-4 (PMC5575036; doi:10.1038/s41598-017-10302-4)
Supplement: Supplementary file 1 — Supplementary Information [file 41598_2017_10302_MOESM1_ESM.docx]

**Supplementary Info**

**A multi-trap microfluidic chip enabling longitudinal studies of nerve regeneration in *Caenorhabditis elegans***

*Sertan Kutal Gokce^1^, Evan Marley Hegarty^2^, Sudip Mondal^2^, Peisen Zhao^1^, Navid Ghorashian^3^*

*Massimo A. Hilliard^4^,* and *Adela Ben-Yakar^1,2,3,5^*

^1^Electrical and Computer Engineering, The University of Texas at Austin, TX 78712

^2^Mechanical Engineering, The University of Texas at Austin, TX 78712

**^3^**Biomedical Engineering, The University of Texas at Austin, TX 78712

^4^ Queensland Brain Institute, The University of Queensland, Brisbane, QLD 4072, Australia

^5^ Institute for Neuroscience, The University of Texas at Austin, TX 78712

**Corresponding author:** [ben-yakar@mail.utexas.edu](mailto:ben-yakar@mail.utexas.edu)

**Supplementary Figures**

**
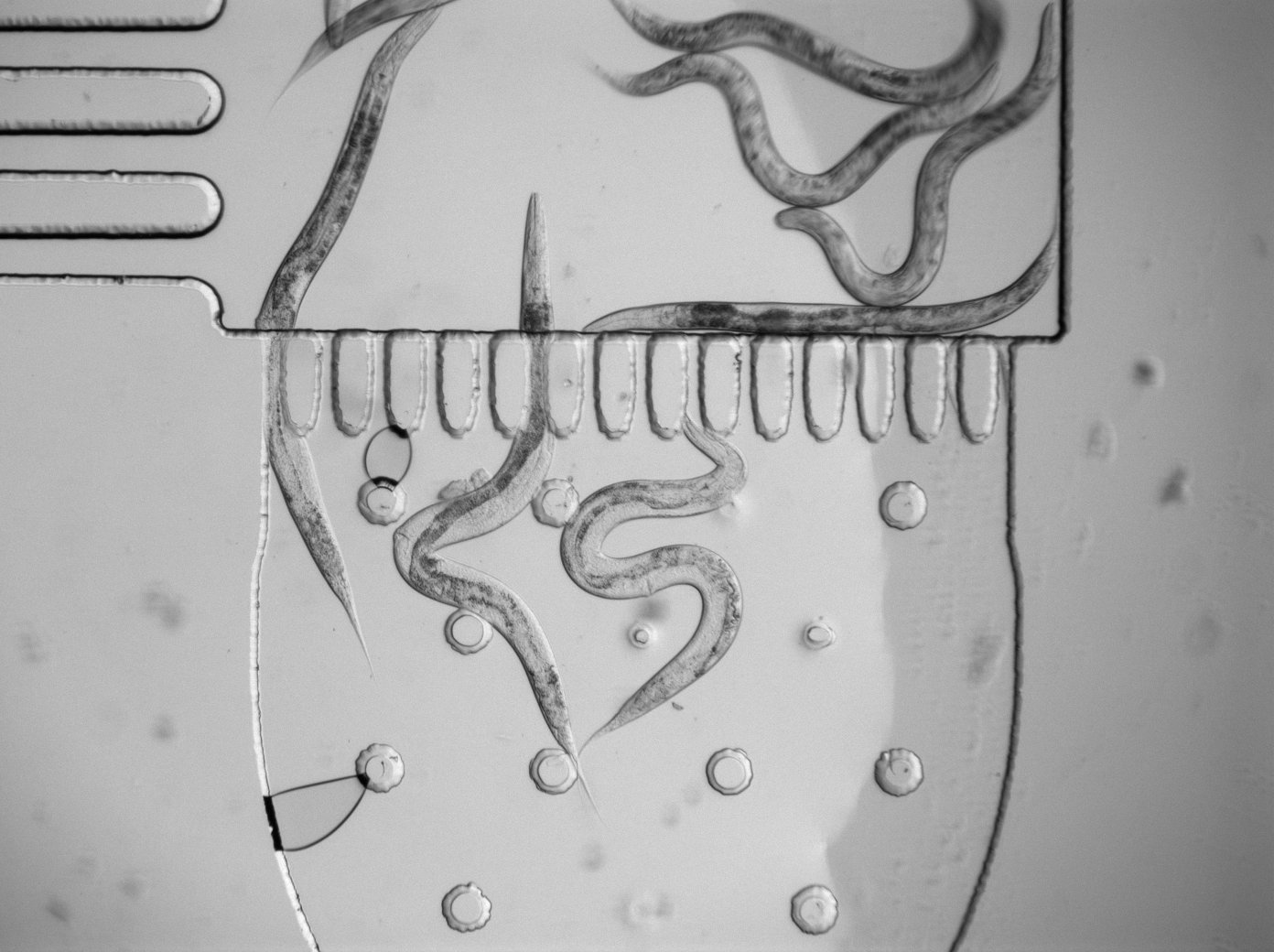
**

**Figure S1: Undesired housing of worms during perfusion and pushing-back process.** In the initial design, the sieve structures were at the same height as the shortest channel height (~20 µm) of immobilization channels. This height caused the animals to get stuck between sieve structures during perfusion and while pushing them back to the housing chamber. In the final design, we incorporated a fourth layer for the sieve structure to be at a shallower ~10 µm height.

**
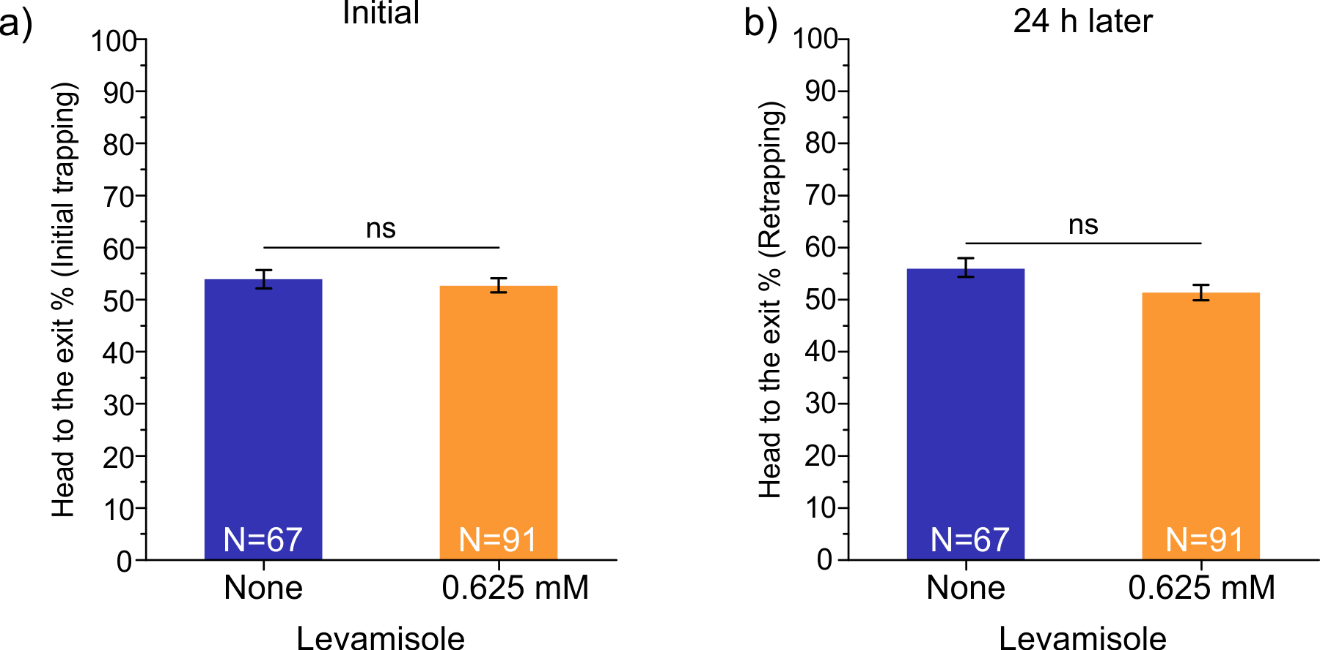
**

**Figure S2: Head-tail orientation characterization.** a) The percentage of the head-tail orientation of worms during initial trapping for the two different on-chip immobilization conditions. For initial trapping for no-levamisole on-chip conditions, 53.9 ± 1.5% of the axotomized worms are immobilized as their heads are in the direction of the exit outlet whereas 52.1 ± 1.5% had the same orientation for 0.625 mM levamisole on-chip immobilization conditions. (Two-tailed t-test; ns, not significant). b) The percentage of the head/tail orientation of worms 24 h after post-surgery recovery for the two different on-chip immobilization conditions. For the initial trapping with no-levamisole on-chip conditions, 55.9 ± 1.8% of the axotomized worms are immobilized with their heads towards the exit outlet whereas 51.5± 1.5% had the same orientation for the 0.625 mM levamisole on-chip immobilization condition (two-tailed *t*-test; ns, not significant). Error bars in (a) and (b) show standard error of mean and the number in each bar indicates number of experiments.


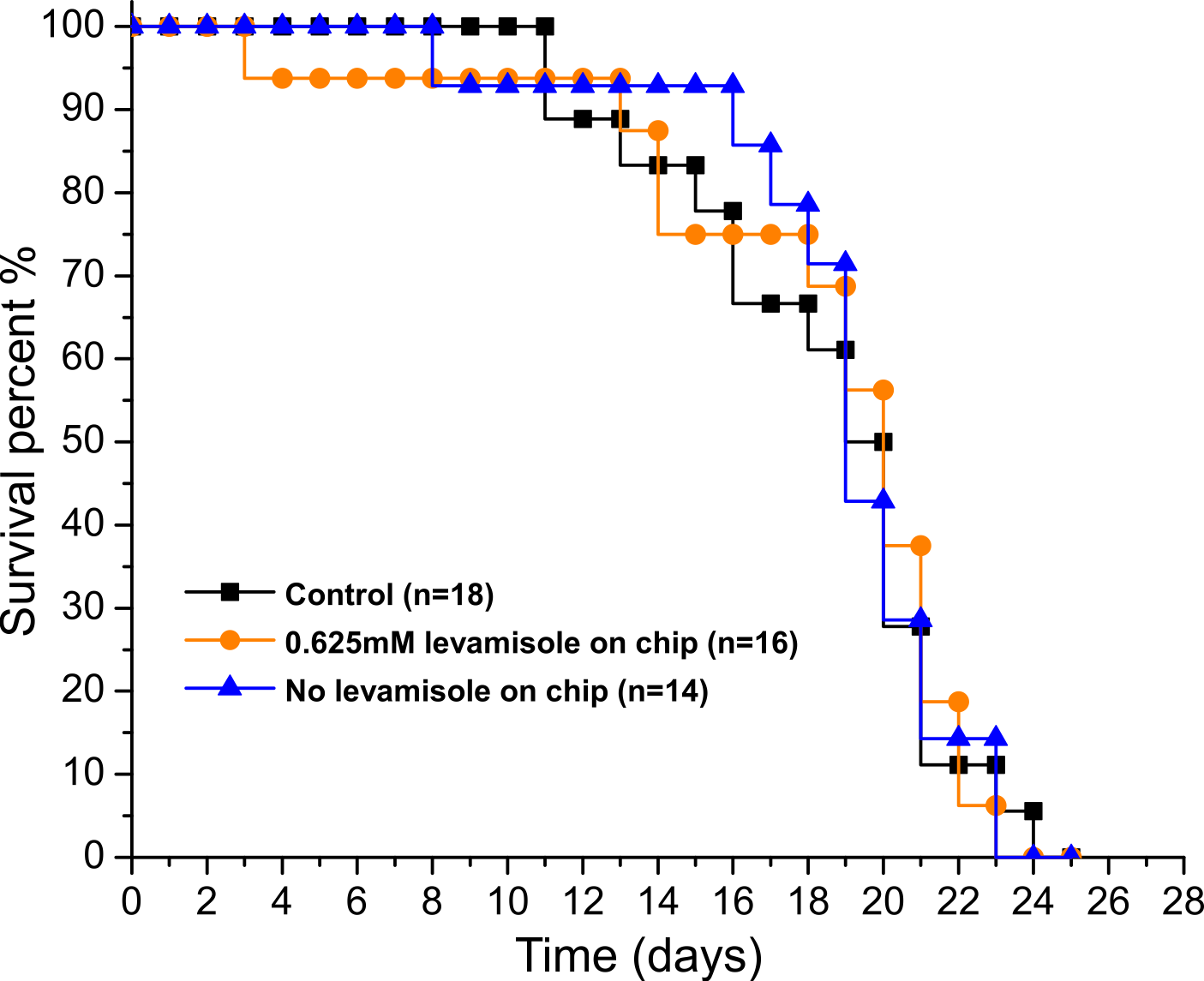


**Figure S3: Lifespan analysis.** The viability of worms immobilized on-chip for two different conditions (without levamisole in blue and 0.625 mM levamisole in orange) and the control group. Number of animals studied is included in the legend. (Log-Rank test, *p* > 0.90, not significant).


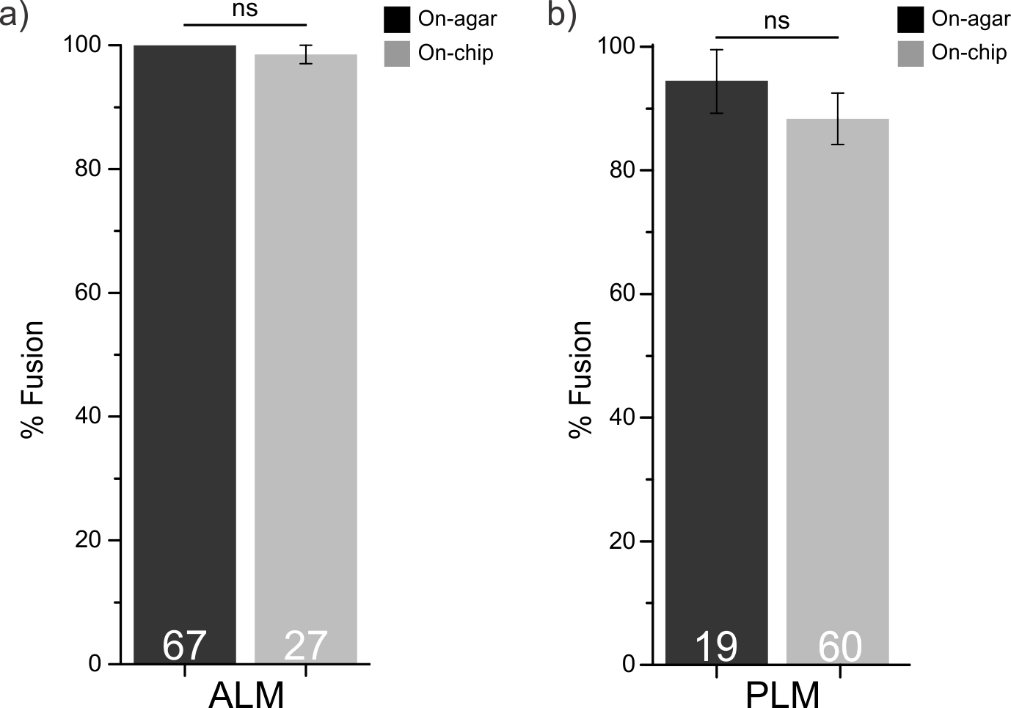


**Figure S4: Effect of on-chip immobilization and housing on axonal fusion:** Quantification of axonal fusion rates of the reconnected animals are shown in (a) and (d) for ALM and PLM neurons, respectively. The error bars show standard error of proportion and the number in each bar indicates the number of animals. We used a two-tailed *t*-test for statistics.

**
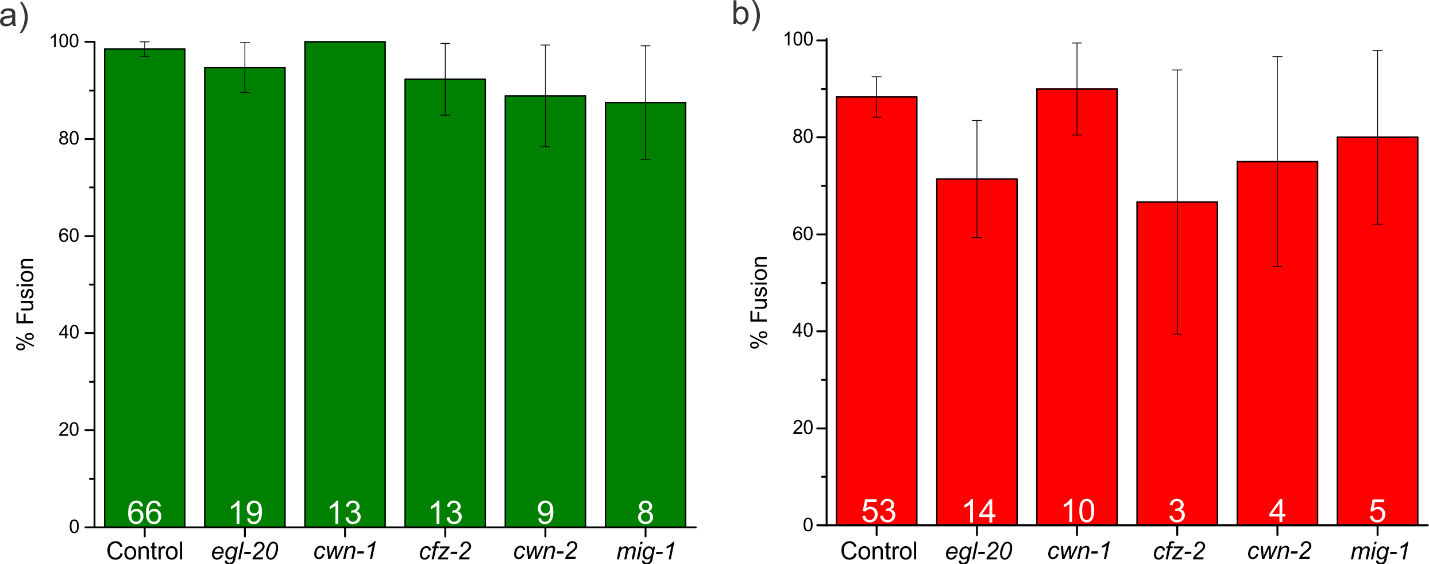
**

**Figure S5: On-chip axonal fusion results:** Quantification of axonal fusion rates among the reconnected animals are shown in (a) and (d) for ALM and PLM neurons, respectively. The error bars show standard error of proportion and the number in each bar indicates the number of animals. We used one-way ANOVA with Dunnet’s correction for multiple comparisons.

**Supplementary Tables**

**Supplementary Table 1:** Comparison of on-chip and on-agar regeneration results for ALM neurons.

| **Genotype** | **Condition** | **Number of animals (N)** | **Regrowth rate%**  **and (N)** | ***p*-value** |
| --- | --- | --- | --- | --- |
| *zdIs5* | On-chip | 81 | 98.8% (80) | *p* = 0.541 |
| *zdIs5* | On-agar | 32 | 100.0% (32) |  |
| **Genotype** | **Condition** | **Number of animals (N)** | **Reconnection rate%**  **and (N)** | ***p*-value** |
| *zdIs5* | On-chip | 80 | 83.7% (67) | *p* = 0.935 |
| *zdIs5* | On-agar | 32 | 84.4% (27) |  |
| **Genotype** | **Condition** | **Number of animals (N)** | **Fusion rate%**  **and (N)** | ***p*-value** |
| *zdIs5* | On-chip | 67 | 98.5% (66) | *p* = 0.531 |
| *zdIs5* | On-agar | 27 | 100.0% (27) |  |
| **Genotype** | **Condition** | **Number of animals (N)** | **Regrowth length (µm)** | ***p*-value** |
| *zdIs5* | On-chip | 13 | 49.7 | *p* = 0.734 |
| *zdIs5* | On-agar | 3 | 55.3 |  |

**Supplementary Table 2:** Comparison of on-chip and on-agar regeneration results for PLM neurons.

| **Genotype** | **Condition** | **Number of animals (N)** | **Regrowth rate%**  **and (N)** | ***p*-value** |
| --- | --- | --- | --- | --- |
| *zdIs5* | On-chip | 97 | 96.9% (94) | *p* = 0.904 |
| *zdIs5* | On-agar | 37 | 97.2% (36) |  |
| **Genotype** | **Condition** | **Number of animals (N)** | **Reconnection rate%**  **and (N)** | ***p*-value** |
| *zdIs5* | On-chip | 94 | 63.8% (60) | *p* = 0.248 |
| *zdIs5* | On-agar | 36 | 52.8% (19) |  |
| **Genotype** | **Condition** | **Number of animals (N)** | **Fusion rate%**  **and (N)** | ***p*-value** |
| *zdIs5* | On-chip | 60 | 88.3% (53) | *p* = 0.416 |
| *zdIs5* | On-agar | 19 | 94.7% (18) |  |
| **Genotype** | **Condition** | **Number of animals (N)** | **Regrowth length (µm)** | ***p*-value** |
| *zdIs5* | On-chip | 26 | 51.8 | *p* = 0.093 |
| *zdIs5* | On-agar | 12 | 64.1 |  |

**Supplementary Table 3:** On-chip axonal regrowth rates of ALM neurons in single mutant animals.

| **Genotype** | **Number of animals (N)** | **Regrowth rate%**  **and (N)** | ***p*-value to *zdIs5*** |
| --- | --- | --- | --- |
| *zdIs5* | 81 | 98.8% (80) |  |
| *cfz-2(ok1201); zdIs5* | 38 | 97.4% (37) | *p* = 0.994 |
| *cwn-1(ok546); zdIs5* | 30 | 96.7% (29) | *p* = 0.976 |
| *cwn-2(ok895); zdIs5* | 40 | 92.5% (37) | *p* = 0.253 |
| *egl-20(n585); zdIs5* | 24 | 95.8% (23) | *p* = 0.931 |
| *mig-1(c1787); zdIs5* | 48 | 95.8% (46) | *p* = 0.831 |

**Supplementary Table 4:** On-chip axonal regrowth rates of PLM neurons in single mutant animals.

| **Genotype** | **Number of animals (N)** | **Regrowth rate%**  **and (N)** | ***p*-value to *zdIs5*** |
| --- | --- | --- | --- |
| *zdIs5* | 97 | 96.9% (94) |  |
| *cfz-2(ok1201); zdIs5* | 34 | 97.1% (33) | *p* = 0.999 |
| *cwn-1(ok546); zdIs5* | 27 | 96.3% (26) | *p* = 0.$998$ |
| *cwn-2(ok895); zdIs5* | 35 | 94.3% (33) | *p* = 0.9596 |
| *egl-20(n585); zdIs5* | 41 | 92.7% (38) | *p* = 0.739 |
| *mig-1(c1787); zdIs5* | 28 | 89.3% (25) | *p* = 0.334 |

**Supplementary Table 5:** On-chip axonal reconnection rates of the regrowing ALM neurons in single mutant animals.

| **Genotype** | **Number of regrowing animals (N)** | **Reconnection rate%**  **and (N)** | ***p*-value to *zdIs5*** |
| --- | --- | --- | --- |
| *zdIs5* | 80 | 83.7% (67) |  |
| *cfz-2(ok1201); zdIs5* | 37 | 35.1% (13) | *p* < 0.001*** |
| *cwn-1(ok546); zdIs5* | 29 | 44.8% (13) | *p* < 0.001*** |
| *cwn-2(ok895); zdIs5* | 37 | 24.3% (9) | *p* < 0.001*** |
| *egl-20(n585); zdIs5* | 23 | 82.6% (19) | *p* = 0.999 |
| *mig-1(c1787); zdIs5* | 46 | 17.4 % (8) | *p* < 0.001*** |

**Supplementary Table 6:** On-chip axonal reconnection rates of the regrowing PLM neurons in single mutant animals.

| **Genotype** | **Number of regrowing animals (N)** | **Reconnection rate%**  **and (N)** | ***p*-value to *zdIs5*** |
| --- | --- | --- | --- |
| *zdIs5* | 94 | 63.8% (60) |  |
| *cfz-2(ok1201); zdIs5* | 33 | 9.1% (3) | *p* < 0.001*** |
| *cwn-1(ok546); zdIs5* | 26 | 38.5% (10) | *p* =0.038* |
| *cwn-2(ok895); zdIs5* | 33 | 12.1% (4) | *p* < 0.001*** |
| *egl-20(n585); zdIs5* | 38 | 36.8% (14) | *p* = 0.006** |
| *mig-1(c1787); zdIs5* | 25 | 20.0% (5) | *p* < 0.001*** |

**Supplementary Table 7:** On-chip axonal fusion rates of the reconnected ALM neurons in single mutant animals.

| **Genotype** | **Number of animals (N)** | **Fusion rate%**  **and (N)** | ***p*-value to *zdIs5*** |
| --- | --- | --- | --- |
| *zdIs5* | 67 | 98.5% (66) |  |
| *cfz-2(ok1201); zdIs5* | 13 | 92.3% (12) | *p* = 0.709 |
| *cwn-1(ok546); zdIs5* | 13 | 100% (13) | *p* = 0.998 |
| *cwn-2(ok895); zdIs5* | 9 | 88.9% (8) | *p* = 0.457 |
| *egl-20(n585); zdIs5* | 19 | 94.7% (18) | *p* = 0.901 |
| *mig-1(c1787); zdIs5* | 8 | 87.5% (7) | *p* = 0.378 |

**Supplementary Table 8:** On-chip axonal fusion rates of the reconnected PLM neurons in single mutant animals.

| **Genotype** | **Number of animals (N)** | **Fusion rate%**  **and (N)** | ***p*-value to *zdIs5*** |
| --- | --- | --- | --- |
| *zdIs5* | 60 | 88.3% (53) |  |
| *cfz-2(ok1201); zdIs5* | 3 | 66.6% (2) | *p* = 0.744 |
| *cwn-1(ok546); zdIs5* | 10 | 90.0% (9) | *p* = 0.999 |
| *cwn-2(ok895); zdIs5* | 4 | 75.0% (3) | *p* = 0.918 |
| *egl-20(n585); zdIs5* | 14 | 71.4% (10) | *p* = 0.351 |
| *mig-1(c1787); zdIs5* | 5 | 80.0% (4) | *p* = 0.981 |

**Supplementary Table 9:** On-chip axonal regrowth lengths of the regrown but not fused ALM neurons in single mutant animals.

| **Genotype** | **Number of animals that regrew but did not fuse (N)** | **Regrowth length (µm)** | ***p*-value to *zdIs5*** |
| --- | --- | --- | --- |
| *zdIs5* | 12 | 49.7 |  |
| *cfz-2(ok1201); zdIs5* | 19 | 39.7 | *p* = 0.407 |
| *cwn-1(ok546); zdIs5* | 12 | 45.2 | *p* = 0.917 |
| *cwn-2(ok895); zdIs5* | 24 | 30.5 | *p* = 0.148 |
| *egl-20(n585); zdIs5* | 5 | 33.5 | *p* = 0.412 |
| *mig-1(c1787); zdIs5* | 34 | 23.0 | *p* = 0.007** |

**Supplementary Table 10:** On-chip axonal regrowth lengths of the regrown but not fused PLM neurons in single mutant animals.

| **Genotype** | **Number of animals that regrew but did not fuse (N)** | **Regrowth length (µm)** | ***p*-value to *zdIs5*** |
| --- | --- | --- | --- |
| *zdIs5* | 26 | 51.8 |  |
| *cfz-2(ok1201); zdIs5* | 28 | 33.2 | *p* = 0.079 |
| *cwn-1(ok546); zdIs5* | 12 | 13.1 | *p* < 0.001*** |
| *cwn-2(ok895); zdIs5* | 25 | 46.5 | *p* = 0.811 |
| *egl-20(n585); zdIs5* | 24 | 28.1 | *p* = 0.015* |
| *mig-1(c1787); zdIs5* | 18 | 21.6 | *p* =0.0012** |

**Supplementary Movies**

**Movie S1.** A real-time video of on-chip trapped worm with no levamisole. The video shows the high-motility of the tail part, hindering the focusing of laser beam on the axon of PLM.

**Movie S2.** A real-time video of on-chip immobilized worm with using 0.625 mM levamisole. The video shows the complete elimination of the tail motility with using a small concentration of levamisole.
